# Impella‐Assisted High‐Risk Percutaneous Coronary Intervention in Cardiogenic Shock With Low‐Flow, Low‐Gradient Aortic Stenosis and Multivessel Coronary Artery Disease: A Case Report

**DOI:** 10.1002/ccr3.73273

**Published:** 2026-07-30

**Authors:** Douni Roger, Waleed Shaker, Zan Siddiqi, George Abela

**Affiliations:** ^1^ University of Michigan/Sparrow Hospital Lansing Michigan USA

**Keywords:** cardiogenic shock, high‐risk percutaneous coronary intervention, Impella 5.5, low‐flow, low‐gradient aortic stenosis, mechanical circulatory support, multivessel coronary artery disease

## Abstract

Patients with cardiogenic shock, low‐flow, low‐gradient aortic stenosis, and multivessel coronary artery disease who are ineligible for surgery present a major therapeutic challenge. Percutaneous mechanical circulatory support may facilitate revascularization in this high‐risk population. A 69‐year‐old man presented after a mechanical fall and was found to have non–ST‐elevation myocardial infarction, acute decompensated heart failure, low‐gradient aortic stenosis, and multivessel coronary artery disease. Echocardiography showed a left ventricular ejection fraction (LVEF) of 25%–30% with mild left ventricular dilation and mildly reduced right ventricular systolic function. Laboratory testing demonstrated elevated troponin, markedly elevated B‐type natriuretic peptide, and acute kidney injury. Chest radiography revealed bilateral pleural effusions and mild atelectasis. Surgical revascularization was not pursued, as the patient was considered at prohibitive operative risk by cardiothoracic surgery. The patient developed cardiogenic shock requiring Impella 5.5 support and subsequently underwent Impella‐assisted high‐risk PCI to the left anterior descending artery. He was successfully weaned from mechanical support, renal function recovered, and guideline‐directed medical therapy was optimized. He was discharged with a wearable cardioverter‐defibrillator and planned repeat echocardiography. This case highlights the role of percutaneous mechanical circulatory support in enabling high‐risk PCI in patients with cardiogenic shock, pseudo‐severe aortic stenosis, and multivessel coronary artery disease when surgical options are not feasible.

## Key Clinical Message

Impella‐assisted high‐risk PCI can provide effective hemodynamic stabilization and allow revascularization in cardiogenic shock due to severe aortic stenosis and multivessel coronary artery disease when surgical revascularization is contraindicated.

## 1 Introduction

Low‐flow, low‐gradient aortic stenosis combined with multivessel coronary artery disease and reduced left ventricular function is associated with poor outcomes, particularly when complicated by cardiogenic shock. Surgical revascularization with valve replacement is often not feasible in patients with excessive operative risk.

In this high‐risk population, percutaneous mechanical circulatory support may facilitate safe revascularization [1].

Percutaneous mechanical circulatory support devices, such as the Impella system, unload the left ventricle and maintain systemic perfusion during complex coronary intervention [2, 3]. This approach may enable high‐risk PCI in patients with cardiogenic shock, severe aortic stenosis, and multivessel coronary artery disease when surgical revascularization is not feasible [4]. However, the use of Impella support in patients with low‐flow, low‐gradient aortic stenosis remains challenging because the device requires transvalvular positioning across a stenotic valve. This raises concerns regarding valve injury, impaired device positioning, and limited flow augmentation in patients with low‐flow, low‐gradient physiology.

Although registry data suggest improved hemodynamic stability with these devices, evidence remains limited in patients with concomitant severe valvular disease [5]. We report a case of Impella 5.5‐supported high‐risk PCI in a patient with cardiogenic shock, multivessel coronary artery disease, and low‐flow, low‐gradient aortic stenosis who was not a surgical candidate.

## 2 Case Presentation

### 2.1 Patient Information

A 69‐year‐old man with ischemic cardiomyopathy (baseline left ventricular ejection fraction [LVEF] 35%–40%), low‐gradient aortic stenosis, hypertension, type 2 diabetes mellitus, and chronic bilateral lower extremity wounds presented after a mechanical fall from a chair, resulting in back pain radiating to the abdomen. He reported progressive lower extremity swelling and pain over 1 month and chronic dental cavities. He denied chest pain, palpitations, syncope, fever, or chills.

### 2.2 Clinical Findings

**FIGURE 1:**
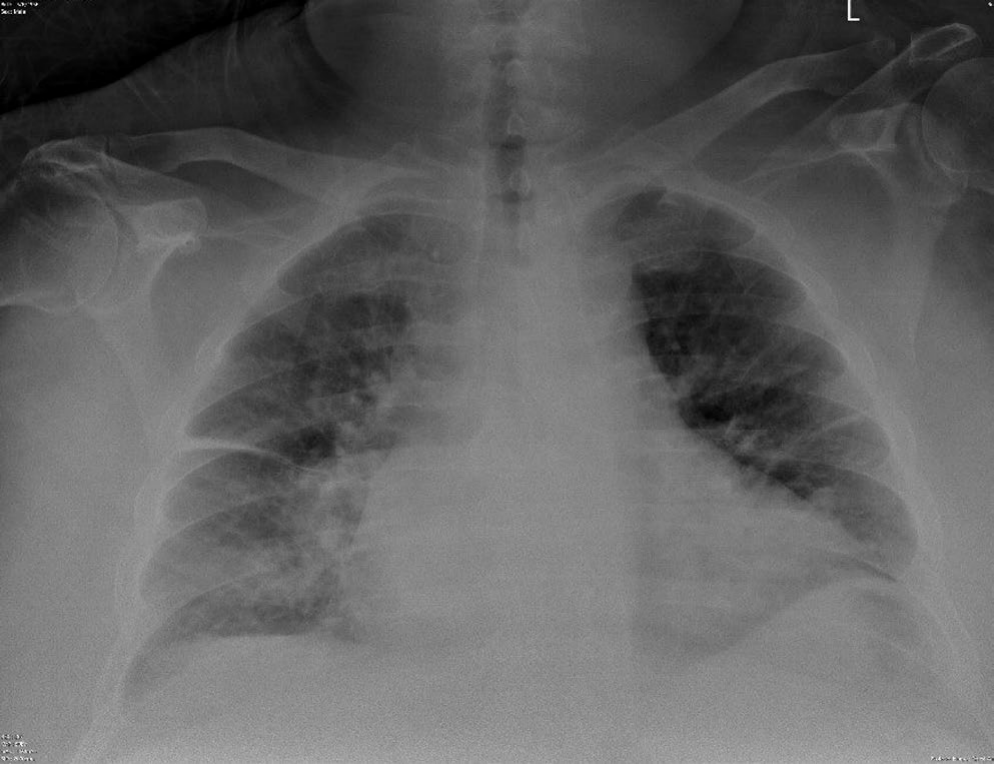
Chest radiograph showed cardiomegaly and findings suggestive of mild acute heart failure, with bilateral pleural effusions (right > left) and mild atelectasis.

On admission, physical examination revealed bilateral lower extremity edema with chronic wounds and signs of volume overload. He developed acute hypoxic respiratory failure and anasarca. Chest radiography (PA and lateral) demonstrated bilateral pleural effusions (right greater than left) and mild atelectasis consistent with acute congestive heart failure (see Figure 1). Electrocardiography showed normal sinus rhythm with premature atrial and ventricular complexes without acute ischemic changes.

### 2.3 Diagnostic Assessment

Laboratory evaluation demonstrated evidence of myocardial injury and cardiorenal syndrome, with worsening renal function and associated electrolyte abnormalities (see Table 1). One of two blood cultures grew coagulase‐negative staphylococci and was considered a contaminant that did not alter cardiovascular management. Venous duplex ultrasonography of the upper and lower extremities was negative for deep vein thrombosis.

**TABLE 1:** Initial labs.

Test	Value	Reference
Troponin (ng/L)	207 → 711	< 14
Potassium (mmol/L)	5.2	3.5–5.0
Sodium (mmol/L)	135	135–145
Creatinine (mg/dL)	1.48	0.7–1.3
BUN (mg/dL)	21	7–20

Transthoracic echocardiography demonstrated a mildly dilated left ventricle with severely reduced systolic function (LVEF 25%–30%), global hypokinesis, moderate left ventricular hypertrophy, biatrial enlargement, and mildly reduced right ventricular systolic function. The right ventricle was moderately dilated with reduced systolic function (TAPSE 1.8 cm). Evaluation of the aortic valve demonstrated low‐flow, low‐gradient aortic stenosis with a dimensionless index of 0.3, peak velocity of 1.62 m/s, and mean gradient of 6 mmHg. Intraprocedural transesophageal echocardiography demonstrated a trileaflet aortic valve with moderate calcification, preserved leaflet opening, and an estimated valve area of approximately 1.5 cm^2^ by planimetry, raising concern for severe aortic stenosis in the setting of severe cardiomyopathy and low‐flow physiology. Additional findings included mild aortic, mitral, tricuspid, and pulmonic regurgitation, large bilateral pleural effusions, and a small pericardial effusion.

Right and left heart catheterization demonstrated markedly elevated filling pressures, low cardiac output, and severe pulmonary hypertension of mixed pre‐ and post‐capillary etiology. Invasive aortic valve assessment using a Langston catheter demonstrated an aortic valve area ranging from 0.99 cm^2^ using thermodilution cardiac output to 1.47 cm^2^ using Fick cardiac output, further supporting low‐flow, low‐gradient physiology with possible severe aortic stenosis.

Coronary angiography demonstrated severe multivessel coronary artery disease, including a critical 99% stenosis of the mid left anterior descending (LAD) artery with bifurcation into two branches exhibiting severe diffuse 70%–80% stenosis. All obtuse marginal branches were severely diseased with approximately 90% stenosis, and the third obtuse marginal branch was chronically totally occluded. The right coronary artery was nondominant with a 90% mid‐segment stenosis. Cardiothoracic surgery deemed the patient a prohibitive candidate for coronary artery bypass grafting because of severe ventricular dysfunction, cardiogenic shock, acute kidney injury, morbid obesity, diabetes mellitus, and chronic bilateral lower extremity wounds.

### 2.4 Clinical Decision‐Making Rationale

The patient underwent multidisciplinary heart‐team evaluation involving interventional cardiology, structural cardiology, heart failure, and cardiothoracic surgery. Coronary angiography demonstrated severe multivessel coronary artery disease with markedly elevated filling pressures, low cardiac output, and mixed pre‐ and post‐capillary pulmonary hypertension [6].

Invasive aortic valve assessment using a Langston catheter demonstrated an aortic valve area ranging from 0.99 cm^2^ using thermodilution cardiac output to 1.47 cm^2^ using Fick cardiac output, raising concern for severe aortic stenosis in the setting of severe cardiomyopathy and low‐flow physiology. Intraprocedural transesophageal echocardiography demonstrated only mild‐to‐moderate valvular calcification with preserved leaflet opening, further supporting severe rather than fixed severe aortic stenosis.

Cardiothoracic surgery deemed the patient a prohibitive surgical candidate because of severe ventricular dysfunction, cardiogenic shock, acute kidney injury, morbid obesity, diabetes mellitus, chronic bilateral lower extremity wounds prohibiting sternotomy, and overall frailty.

Mechanical circulatory support with Impella 5.5 was selected over Impella CP because of the patient's markedly elevated filling pressures, pulmonary hypertension, severe cardiomyopathy, body habitus (BMI 39 kg/m^2^), and anticipated need for prolonged hemodynamic support during aggressive diuresis and staged high‐risk PCI. Alternative strategies including balloon aortic valvuloplasty, transcatheter aortic valve replacement, intra‐aortic balloon pump support, and venoarterial extracorporeal membrane oxygenation were considered. Impella support was favored because of its ability to directly unload the left ventricle while augmenting cardiac output without increasing left ventricular afterload.

The left anterior descending artery was prioritized for revascularization because it represented the most prognostically significant lesion in the setting of ischemic cardiomyopathy and cardiogenic shock. Additional intervention on obtuse marginal branches was deferred because the vessels were diffusely diseased and unlikely to significantly improve heart failure outcomes.

### 2.5 Therapeutic Intervention

The patient developed progressive cardiogenic shock with worsening end‐organ dysfunction and was transferred to the intensive care unit for mechanical circulatory support. Following multidisciplinary heart‐team discussion, an Impella 5.5 device was placed via a right axillary approach on hospital day 8 under transesophageal echocardiographic guidance. Intraprocedural imaging confirmed appropriate positioning of the device across the aortic valve with the inflow portion measuring approximately 4.7 cm below the aortic valve annulus within the left ventricle and the outflow portion visualized in the ascending aorta without evidence of aortic injury, dissection, or pericardial effusion.

Blood pressure was managed with clevidipine infusion targeting a systolic blood pressure of 100–120 mmHg. Aggressive diuresis was achieved using continuous intravenous furosemide infusion with adjunctive acetazolamide, resulting in significant urine output exceeding 4.8 L/day and progressive improvement in anasarca and respiratory status. Lactate levels normalized and mixed venous oxygen saturation improved to 83%, suggesting improved systemic perfusion and hemodynamic stabilization. Anticoagulation with intravenous heparin was temporarily withheld because of groin‐site hematoma and hematuria. A bicarbonate purge solution was utilized for Impella support. Linezolid was administered for 5 days for suspected lower extremity cellulitis.

Following optimization of volume status and hemodynamics, the patient underwent Impella‐assisted high‐risk PCI. Ultrasound‐guided right femoral arterial access was obtained, and intravascular ultrasound‐guided PCI of the left anterior descending artery was successfully performed using overlapping 2.5 × 38 mm and 2.25 × 12 mm Synergy drug‐eluting stents. Predilatation was performed with compliant and noncompliant balloons prior to stent deployment. Intravascular ultrasound demonstrated well‐expanded and well‐apposed stents without significant edge dissection or perforation. Final angiography demonstrated TIMI 3 flow with 0% residual stenosis in the LAD.

Attempts to wire and treat additional diseased LAD branches and diagonal vessels were limited by difficult vessel angulation and procedural complexity. Revascularization strategy was intentionally simplified because of increasing radiation exposure and the need to minimize procedural duration in the setting of cardiogenic shock. Additional intervention involving severely diseased obtuse marginal branches was deferred because these lesions were considered less likely to improve heart failure outcomes and could be reassessed in the outpatient setting if clinically indicated.

The patient remained hemodynamically stable throughout the procedure without evidence of device‐related complications. The Impella device was maintained for continued postprocedural support with plans for removal following stabilization.

Guideline‐directed medical therapy for ischemic cardiomyopathy and heart failure with reduced ejection fraction was initiated and titrated following hemodynamic stabilization, including metoprolol succinate transitioned to carvedilol, sacubitril/valsartan, dapagliflozin, spironolactone, aspirin, ticagrelor, and high‐intensity statin therapy.

### 2.6 Outcome and Follow‐Up

The Impella device was removed after hemodynamic stabilization. Respiratory failure resolved, urine output exceeded 7 L/day during diuresis, and renal function improved (creatinine decreased from 1.60 to 1.29 mg/dL). He was weaned from intravenous antihypertensive therapy and transitioned to oral diuretics. Table 2 outlines the chronological sequence of clinical events during the patient's hospitalization.

**TABLE 2:** Sequence of clinical events.

Date	Event
12/12	Hospital admission following a fall
12/18	Coronary angiography performed
12/20	Impella device placement
12/22	Percutaneous coronary intervention to the left anterior descending artery
12/23	Impella device removed
12/24	Transfer to the general medical floor

The patient remained hemodynamically stable and was transferred to the medical ward. Physical and occupational therapy recommended subacute rehabilitation. Due to persistent left ventricular dysfunction and recent cardiogenic shock, a wearable cardioverter‐defibrillator (LifeVest) was provided prior to discharge.

At discharge, he was clinically stable on optimized guideline‐directed medical therapy, including carvedilol, sacubitril/valsartan (dose adjusted for renal function), spironolactone, dapagliflozin, aspirin, ticagrelor, and atorvastatin. A repeat transthoracic echocardiogram was planned for 3 months.

## 3 Discussion

This case highlights the complexity of managing cardiogenic shock in the setting of multivessel coronary artery disease and low‐flow, low‐gradient aortic stenosis with features concerning for pseudo‐severe AS.

Despite these challenges, Impella support may provide substantial physiologic benefit in carefully selected patients. By directly unloading the left ventricle, reducing left ventricular end‐diastolic pressure, augmenting cardiac output, and improving coronary perfusion pressure, Impella may stabilize systemic perfusion while reducing myocardial oxygen demand. Contemporary observational data and recent cardiogenic shock experiences have demonstrated improved hemodynamic stabilization and feasibility of complex revascularization strategies with Impella‐supported PCI in selected high‐risk populations [6, 7]. Compared with intra‐aortic balloon pump support, Impella provides greater forward flow and ventricular unloading. In contrast to venoarterial extracorporeal membrane oxygenation, which may increase left ventricular afterload, Impella offers direct ventricular decompression.

In the present case, the patient demonstrated improved urine output, normalization of lactate, stabilization of end‐organ perfusion, and successful tolerance of high‐risk PCI following Impella 5.5 insertion. The device was successfully positioned using a right axillary approach under transesophageal echocardiographic guidance without evidence of aortic injury, significant valvular compromise, or pericardial effusion.

This case demonstrates the feasibility of Impella‐assisted PCI in cardiogenic shock complicated by severe aortic stenosis and multivessel coronary artery disease. Mechanical circulatory support unloads the left ventricle, improves coronary perfusion, and stabilizes systemic circulation, enabling complex PCI [2, 3]. Registry data have shown improved short‐term hemodynamic outcomes in cardiogenic shock [1, 4, 6, 7]. This patient's course also highlights the importance of managing cardiorenal syndrome and optimizing guideline‐directed medical therapy following stabilization [5].

## 4 Conclusion

This case suggests that Impella 5.5‐supported high‐risk PCI may be feasible in carefully selected patients with cardiogenic shock, low‐flow, low‐gradient aortic stenosis with pseudo‐severe physiology, and multivessel coronary artery disease who are not surgical candidates. Early mechanical circulatory support may facilitate ventricular unloading, improve end‐organ perfusion, and enable successful revascularization while allowing optimization of guideline‐directed medical therapy.

## 5 Limitations

This report has several limitations. As a single‐patient experience, the findings are hypothesis‐generating and may not be generalizable to broader populations with cardiogenic shock and low‐flow, low‐gradient aortic stenosis. Serial invasive hemodynamic measurements following Impella implantation were limited, and long‐term follow‐up data were unavailable. In addition, selection bias and survivor bias are inherent limitations of case reports. Comparative conclusions regarding superiority over alternative mechanical circulatory support strategies cannot be made.

## Author Contributions

**Douni Roger:** conceptualization, data curation, methodology, project administration, resources, software, writing – original draft, writing – review and editing. **Waleed Shaker:** investigation, resources, writing – review and editing. **Zan Siddiqi:** supervision. **George Abela:** conceptualization, resources, supervision.

## Funding

The authors have nothing to report.

## Consent

Written informed consent was obtained from the patient for the publication of this case report and any accompanying images or clinical information. The patient was informed that all reasonable efforts would be made to ensure anonymity and that no identifying information would be disclosed.

## Data Availability

The data supporting the findings of this study are available within the article. Additional de‐identified clinical data are available from the corresponding author upon reasonable request, subject to institutional policies and protection of patient confidentiality.
